# Human Activity Recognition Based on Symbolic Representation Algorithms for Inertial Sensors

**DOI:** 10.3390/s18114045

**Published:** 2018-11-20

**Authors:** Wesllen Sousa Lima, Hendrio L. de Souza Bragança, Kevin G. Montero Quispe, Eduardo J. Pereira Souto

**Affiliations:** Instituto de Computação, Universidade Federal do Amazonas, Manaus CEP 69067-005, Brazil; hendrio.luis@gmail.com (H.L.d.S.B.); kevingmq@gmail.com (K.G.M.Q.); esouto@icomp.ufam.edu.br (E.J.P.S.)

**Keywords:** human activity recognition, representation symbolic algorithms, inertial sensors

## Abstract

Mobile sensing has allowed the emergence of a variety of solutions related to the monitoring and recognition of human activities (HAR). Such solutions have been implemented in smartphones for the purpose of better understanding human behavior. However, such solutions still suffer from the limitations of the computing resources found on smartphones. In this sense, the HAR area has focused on the development of solutions of low computational cost. In general, the strategies used in the solutions are based on shallow and deep learning algorithms. The problem is that not all of these strategies are feasible for implementation in smartphones due to the high computational cost required, mainly, by the steps of data preparation and the training of classification models. In this context, this article evaluates a new set of alternative strategies based on Symbolic Aggregate Approximation (SAX) and Symbolic Fourier Approximation (SFA) algorithms with the purpose of developing solutions with low computational cost in terms of memory and processing. In addition, this article also evaluates some classification algorithms adapted to manipulate symbolic data, such as SAX-VSM, BOSS, BOSS-VS and WEASEL. Experiments were performed on the UCI-HAR, SHOAIB and WISDM databases commonly used in the literature to validate HAR solutions based on smartphones. The results show that the symbolic representation algorithms are faster in the feature extraction phase, on average, by 84.81%, and reduce the consumption of memory space, on average, by 94.48%, and they have accuracy rates equivalent to conventional algorithms.

## 1. Introduction

Recently, the rapid dissemination of smartphones with sensing capabilities has enabled the advancement of the Human Activity Recognition (HAR) area. This advancement has benefited applications such as health care [1,2], monitoring physical activities [3,4], domestic activities [5], and safety [6,7]. As a result, many solutions have been proposed based on machine learning algorithms, especially the shallow algorithms (e.g., SVM, Decision Tree, Naive Bayes and KNN) [4,8,9] and, more recently, the deep learning algorithms based on neural networks (e.g., CNN, RNN, RBM, SAE, DFN and DBM) [10,11,12]. The main difference between the two approaches lies in the way the data is prepared. Shallow algorithms, for example, rely on human knowledge about the problem domain to manually configure the data segmentation and reduction of data noise steps, and especially the feature extraction and feature selection steps. On the other hand, deep algorithms are able to segment data, reduce noise, and extract relevant features automatically [10]. This has increased the efficiency of classification models and reduced human influence on HAR solutions.

Considering the limited hardware capacity of smartphones, the main problem of both approaches is still the high consumption of computational resources related to memory and processing, especially when it comes to deep neural networks. In addition, smartphones still have limited power resources, and high-cost solutions tend to drain the battery faster. Deep neural networks, for example, require a large processing capacity during the training of classification models. For this reason, the Graphic Processing Units (GPUs) [12] available on desktop computers are used. Thus, deep learning algorithms become impractical for the development of online applications that run completely on smartphones and are not dependent on external processing (e.g., servers) [13]. For this reason, this work focuses only on the evaluation of strategies based on shallow algorithms with the objective of proposing low-cost online solutions capable of running completely on the smartphone.

In the context of shallow algorithms, the computational cost of the solutions is concentrated in the feature extraction step. The feature extraction step is crucial for generating accurate classification models. This step consists of transforming raw sensor data into useful and compact information in order to facilitate the learning process of machine learning algorithms. For example, in the case of deep neural networks, the features are represented by the neurons of the hidden layers. In the case of shallow algorithms, the features are divided into domains of representation such as time and frequency [14]. Time domain features are extracted by means of mathematical functions used to extract statistical information from the data. Frequency domain features are extracted by mathematical functions that capture repetitive patterns of data. In an attempt to reduce computational cost in the feature extraction step, Khan et al. [15] proposed the use of only the time domain features. However, the use of many features generates databases with high dimensionality, influencing the processing cost of the classification model’ training step. In addition, the use of low cost classification algorithms (e.g., Naive Bayes and Decision Tree), elimination of the steps of data noise reduction, and feature selection also reduce the cost of solutions [13].

In addition to the time and frequency domain features, the HAR literature also cites the existence Discrete domain features are extracted by means of symbolic representation algorithms like Symbolic Aggregate Approximation (SAX) [16] and Symbolic Fourier Approximation (SFA) [17]. These algorithms have discretization functions that transform data segments into symbols. The frequency of the symbols can evidence repetitive patterns in the data. The advantages of discrete domain features include the ability to reduce the data dimensionality and data numerosity during the feature extraction step. In the other domains, the data dimensionality reduction occurs only after the feature extraction step with the application of methods such as information gain [18] and PCA [15]. The fact is that the discrete domain reduces the data dimensionality in a natural way, since the symbols are compact representations of the data segments. In addition, the feature extraction process in the discrete domain is considered a natural reducer of data noise in time series [16,17]. From the perspective of the other domains features it is necessary to use extra methods such as Lowpass [19] and moving average [20] filters. In practice, the use of the discrete domain features implies a reduction in the execution of unnecessary steps of the standard HAR methodology.

In short, symbolic representation algorithms allow a massive amount of data to be reduced to a reasonable and representative number of symbols [21]. This contributes to reducing the complexity and computational cost of HAR solutions, opening doors for the use of new HAR strategies in the context of smartphones with limited memory and processing resources. In relation to the learning of symbolic data, there are specific classification algorithms that can be used in the classification models training step. Some of them are extensions adapted to the SAX and SFA algorithms such as the SAX-Vector Space Model (SAX-VSM) [22], Bag-of-SFA Symbols (BOSS) [23], BOSS-Vector Space (BOSS-VS) [24], and Word Extraction for Time Series Classification (WEASEL) algorithms [25]. However, these algorithms cannot handle time series with multiple coordinates. For this, it is necessary that all of them be adapted so that they can handle the three-dimensional inertial sensors data.

In this context, this article has two main contributions. The first one is the evaluation of SAX and SFA strategies and their extensions SAX-VSM, BOSS-VS, BOSS and WEASEL with the purpose of introducing the techniques of symbolic representation in the HAR area. The second is to propose an adaptation to the SAX-VSM, BOSS-VS, BOSS-Model and WEASEL algorithms so that they become able to handle multiple time series. The objective of this article is to improve HAR solutions based on smartphones instrumented with inertial sensors with the use of scalable and low cost strategies. The experiments were performed on three databases UCI-HAR [26], WISDM [27] and SHOAIB [28]. These databases are commonly used in the literature to validate HAR solutions. The results show that the symbolic representation algorithms are more accurate than the shallow algorithms, that they are on average 84.81% faster in the feature extraction step, and that they reduce on average 94.48% of the memory space consumption.

This paper is organized as follows: Section 2 presents the concepts related to the symbolic representation of the data. Section 3 discusses the discretization algorithms and the process of feature extraction of the discrete domain. Section 4 presents the classification algorithms adapted to manipulate discrete domain features. Section 5 presents the proposals for adapting the symbolic classification algorithms to work with the multidimensional time series. Section 6 presents the evaluation of classification models based on symbolic representation algorithms. Section 7 presents the work related to this research. Finally, Section 8 presents the conclusions of this work.

## 2. Features Representation in the Discrete Domain

**Figure 1:**
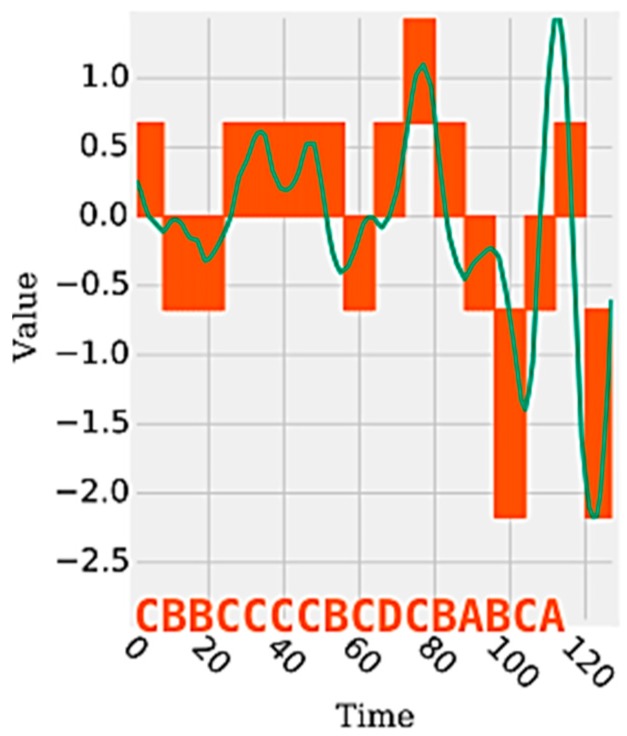
Example of a discretized time series [29].

The features of the time domain and frequency domain are represented by continuous values extracted by statistical and mathematical functions applied on a set of segments of a time series. Such functions can be used to generate numerous attributes (e.g., mean, standard deviation, and variance) from the data signals generated by the sensors, which together are able to represent and discriminate a human activity. On the other hand, the data representation in the discrete domain has, as a feature, a single attribute represented by a sequence of symbolic values. Each symbol represents a range of continuous values contained in a given segment of the signal. Figure 1 shows an example of a time series segment composed of 140 samples and represented by the word (or symbol) “cbbccccbcdcbabca”. Each word is formed by a set of letters limited by an alphabet. A word represents a numerical approximation of a subsequence composed of continuous values. Therefore, the discrete domain features are represented by a set of words, related to each other, capable of discriminating a human activity from the signals extracted from the inertial sensors.

**Figure 2:**
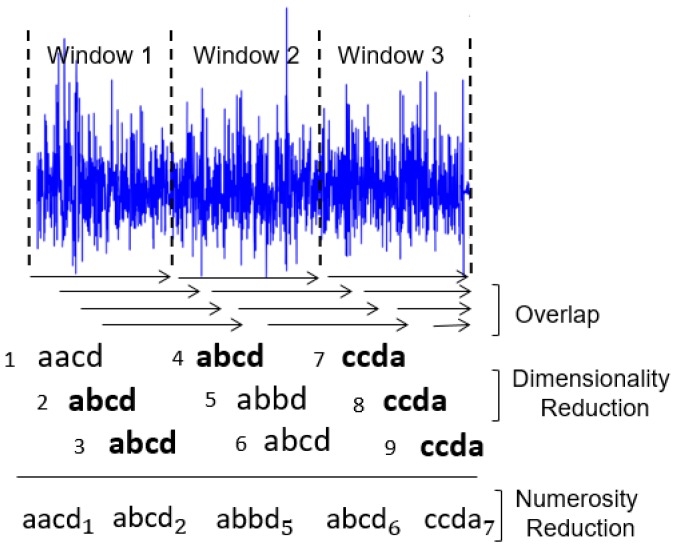
Example of dimensionality and numerosity reduction in a discretized time series represented by words. In this case, the numbers indicate the indexes and the bold words indicate the repeated words.

During the time series discretization, the words are organized sequentially. For each of them are assigned indexes that represent the original time series positions in which each word belongs. This sequential organization of indexed words allows reducing the amount of data after the discretization process. If there are repeated words organized sequentially, the data can be reduced further through the numerosity reduction technique. This technique adds another competitive advantage to the discrete domain features, increasing the advantage of data dimensionality reduction. Numerosity reduction is possible because the indexes represent each word positions in the original time series. In other words, given a subsequence of repeated words, only the first word must be retained and the remaining words can be deleted [30]. Figure 2 shows an example of how dimensionality and numerosity reduction occurs in a discretized time series.

Although there are risks of information loss in the discretization process, there is a mathematical proof, demonstrated by Lin et al. [31] and Schäfer and Högqvist [17], which ensures that the distances between two discrete time series are equivalent to the distances of the same time series in their original state. This mathematical proof refers to the calculation of the lower bounding between raw time series and discretized time series. In addition, the calculation of the distance between two subsequences is considerably faster in the discretized series. On the other hand, there is no loss of information in the numerosity reduction process since the sequence of the omitted symbols can be easily retrieved by correctly manipulating the symbol position indexes.

## 3. Discretization

This section presents two symbolic representation algorithms, SAX and SFA, which can be used to discretize the inertial sensors’ data. These algorithms are applied over the time windows defined in the segmentation step.

### 3.1. SAX—Symbolic Aggregate Approximation

SAX is a symbolic discretization algorithm in which symbols are represented by words [16]. Each word is represented by a set of letters belonging to an alphabet of limited size. The SAX consists of 4 steps: The first step deals with a normalization of the time series. The normalization step consists of a process in which the values of the mean, u, and the standard deviation, σ, of the segments should be around 0 and 1, respectively. Equation (1) shows the mathematical formula used in normalization:

(1) $$x_{i}^{\prime }=\frac{x_{i}-u}{\sigma }$$

where $x_{i}$ represents the time series value at position i and $x_{i}^{\prime }$ is the result of normalization.

The second step deals with the application of the Piecewise Aggregate Approximation (PAA) numerical approximation algorithm [32] to reduce the data dimensionality. In this case, the sequences are divided into subsequences M of equal size, where each subsequence is represented by the mean of their points. This approach makes SAX a natural data noise reducer equivalent to moving average filters. Equation (2) shows the mathematical formula of the PAA applied in a subsequence:

(2) $${\bar{x}}_{i}=\frac{M}{n}\overset{\left(n/M\right)i}{\underset{j=\frac{n}{M\left(i-1\right)}+1}{\sum }}x_{j}$$

where M is the number of subsequence values, n is the number of sequence values, $x_{j}$ is a given value at position j and ${\bar{x}}_{i}$ is the mean of a given sequence. Each subsequence defined by the PAA represents a letter. In this way, the number of subsequences contained in a sequence represents the number of letters of a word.

**Figure 3:**
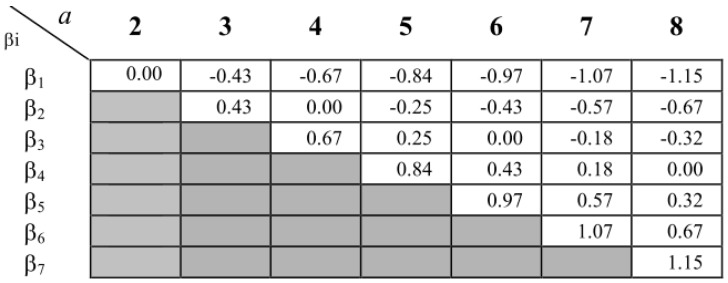
Statistical table used in the definition of breakpoints [33].

The third step deals with the definition of the letters through the limited alphabet size. Each letter is defined by a boundary interval known as breakpoints. These breakpoints are defined by $B=\left(\beta_{1},\beta_{2},\ldots ,\beta_{n}\right)$, such as $\beta_{i-1}<\beta_{i}$, $\beta_{1}>-\infty$ and $\beta_{n}<\infty$. The values of $\beta_{s}$ are defined by means of a statistical table that divides the Gaussian Curve, derived from normalization, into equal areas. Each area is delimited by the interval $\left[B_{j-1},B_{j}\right)$. In other words, breakpoints form intervals in which the first and last point represent −∞ and +∞. Figure 3 shows the statistical table used in the definition of breakpoints.

**Figure 4:**
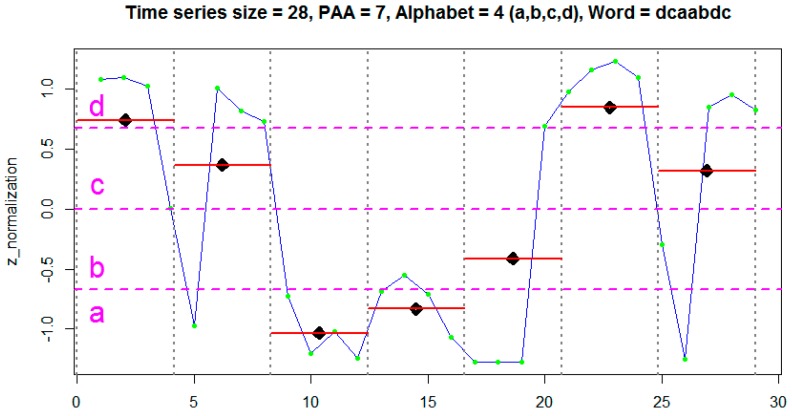
Example of time series discretization with SAX.

Finally, the last step is responsible for the generation of the words based on the letters concatenation generated in the previous step. Figure 4 shows an example of an application of the SAX algorithm on a sequence containing 30 samples (x-axis). As can be observed, samples’ normalization resulted in a Gaussian distribution with amplitude values varying in an open range between −1.5 and 1.5. The samples were divided into 7 subsequences of equal size by the PAA algorithm (vertical lines), and at 4 intervals by the breakpoints (horizontal lines). In this way, this subsequence is represented by a word with 7 letters formed by an alphabet of size 4 (a, b, c, d). In this case, the word equivalent to this subsequence is “dcaabdc”.

Shieh and Keogh [33] proposed an evolution of the SAX algorithm called Indexable Symbolic Aggregate approximation (iSAX). iSAX was developed for the purpose of eliminating the manual setting of the input parameter for the alphabet size. Elimination of this parameter allows the iSAX to be able to represent a subsequence by means of several words with different resolutions, that is, words composed of alphabets of different sizes. It means that the higher the word resolution, the greater is the approximation of the symbolic data with the original data subsequence.

### 3.2. SFA—Symbolic Fourier Approximation

SFA is a symbolic representation algorithm that also uses words composed by an alphabet of limited size [17]. The main differences between SFA and SAX are: (a) the SFA uses the Discrete Fourier Transform (DFT) instead of the PAA; (b) the normalization of the data in the SFA is optional; and (c) the SFA has a technique, called Multiple Coefficient Binning (MCB), to define the breakpoints’ positions.

**Figure 5:**
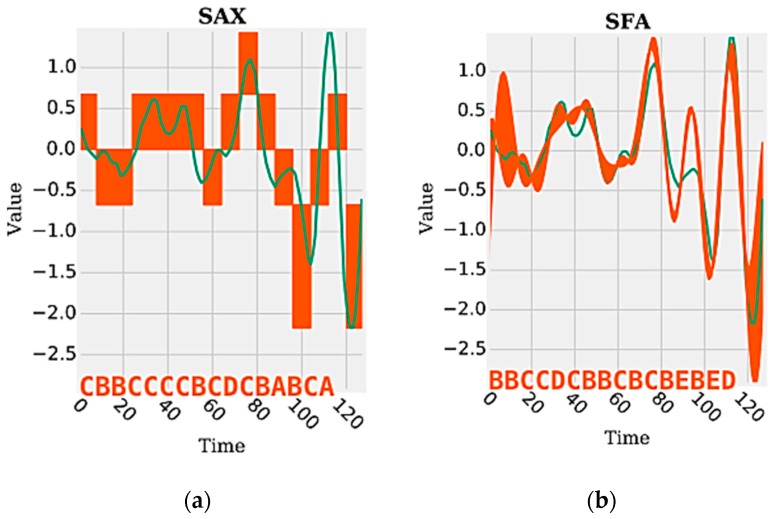
Visual example of the difference between SAX (**a**) and SFA (**b**) [29].

Figure 5 shows a visual example of the difference between the SAX and the SFA. As can be observed, the SFA visually has greater ability to approximate the original subsequence. This fact is proven by Schäfer and Högqvist [17] by calculating the distance between the SFA and the SAX/iSAX. The results show that the similarity between two words generated by SFA has fewer false alarms compared to the similarity between two words generated by SAX/iSAX. This is possible because the DFT has greater approximation capacity (statistically more significant) when compared to the PAA.

**Figure 6:**
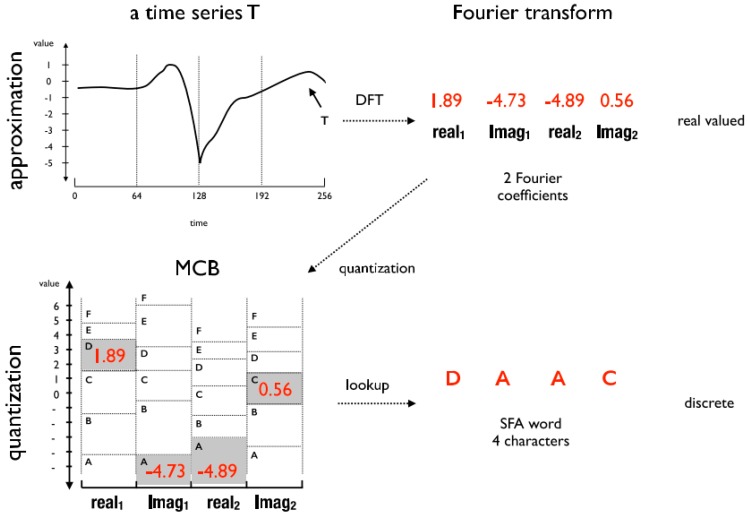
Subsequence T is approximated using the DFT and quantized using the MCB to finally generate the word ‘DAAC’ of size 4 and alphabet of size 6 [29].

The SFA algorithm is divided into two phases: approximation and quantization. As shown in Figure 6, the approximation phase consists of the original subsequence transformation into another subsequence composed of the real and imaginary coefficients extracted from the DFT. This phase also works as signal noise reduction, since the coefficients extracted from the DFT are equivalent to the lowpass filter [17]. In order to optimize the calculation of the coefficients, the SFA uses a modified version of the DFT called the Momentary Fourier Transform (MFT). The main difference between one and the other is that the MFT is able to calculate the coefficients in real time, recursively, based on the previous intervals [29].

**Figure 7:**
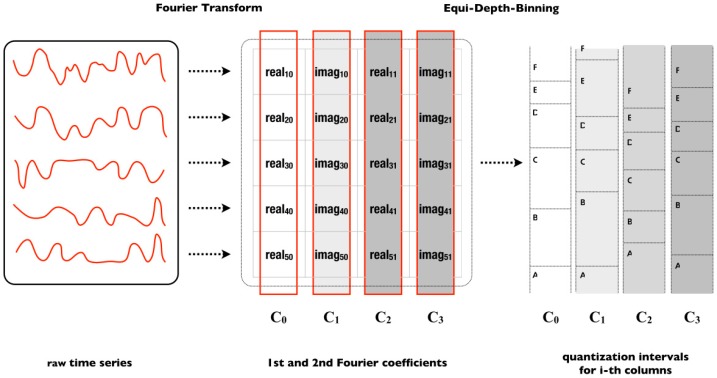
Example of generation of the MCB by means of the coefficients of the MFT [29].

The quantization phase consists of the application of the MCB technique with the purpose of defining the best breakpoints’ positions that delimit the regions’ intervals for each alphabet letter. Basically, the subsequences used to generate the MCB are organized in a matrix in which the real and imaginary coefficients are extracted by the MFT. Thus, each coefficient represents a breakpoint. Two breakpoints represent an interval also called bin. Among the coefficients generated by the MFT, not all are used, since the number of coefficients depends on the word and alphabet sizes passed as a parameter for the SFA. For example, Figure 7 shows a visual example of how the MCB is structured.

Formally, the MCB is represented by a matrix $A=\;{\left(a_{ij}\right)}_{i=1,\;\ldots ,N;j=1,\;\ldots ,\;l}$, where the values of $a_{ij}$ represent the first coefficients of the Fourier transform over the values of the subsequences $T_{i}$, where i ∈[1, …, N]. Thus, matrix A is represented as follows:

$$A=\left(\begin{array}{c} MFT\left(T_{1}\right)\\ MFT\left(T_{i}\right)\\ MFT\left(T_{N}\right) \end{array}\right)=\left(\begin{array}{ccc} real_{11}\;img_{11} & \cdots & real_{12}\;img_{12}\\ real_{i1}\;img_{i1} & \cdots & real_{i2}\;img_{i2}\\ real_{N1}\;img_{N1} & \cdots & real_{N2}\;img_{N2} \end{array}\right)=\left(C_{1},\;\ldots ,\;C_{N}\right)$$

where $C_{N}$ represents the n-th column of the coefficients matrix with real and imaginary values. Thus, each column $C_{j}\;\in A$, where j ∈[0…N), represents a symbol belonging to a finite alphabet $\Sigma =\left\{symbol_{1},\;\ldots ,\;symbol_{c}\right\}$ of size c+1. Each symbol of the alphabet Σ is defined by the intervals formed by the ordered coefficients of column $C_{j}$. The intervals are represented by $\beta_{j}\left(0\right)<\;\ldots \;<\;\beta_{j}\left(c\right)$, where j ∈[0…N), $\beta_{i}\left(0\right)=\;-\infty$ and $\;\beta_{i}\left(c\right)=\;+\infty$. Therefore, the bins of the quantization process are defined as:

$$Bin_{j}\left(i\right)=\left[\beta \left(i-1\right),\;\beta \left(i\right)\right),\;j\in \left[0\ldots N\right),\;i\;\epsilon \;\left[1\ldots C\right]$$

Each bin is labeled with the i-th symbol of the alphabet as follows:

$$Bin_{j}\left(i\right)≜\;symbol_{a}\in \;\Sigma$$

**Figure 8:**
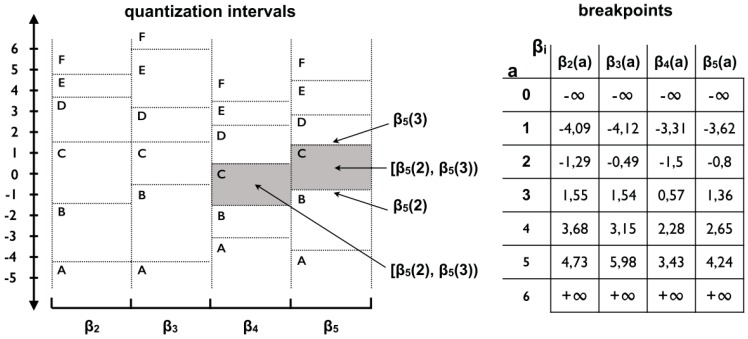
Visual illustration of the distribution of bins and breakpoints in an MCB. In this case, the word size is 4 and the alphabet is 6 [29].

The example in Figure 7 shows the generation of an MCB from five sequences. The input parameters defined were: 4 for the word size and 6 for the alphabet size. Therefore, to satisfy word size, the first 4 coefficients of all sequences were selected (columns) and, to satisfy the alphabet size, the coefficients of each column were ordered in ascending order and then the first 7 rows (*c* + 1) of coefficients were selected. The resulting coefficient matrix forms the following bins depicted in Figure 8.

Unlike the SAX/iSAX algorithm, the SFA generates bins with different intervals for each column of coefficients. This behavior considerably reduces the risks of information loss, so that this strategy is able to adapt better to the data variation. In addition, the SFA always chooses the first coefficients because these coefficients capture the main frequency components, and the remaining components do not affect the accuracy of the approximation significantly [17]. The main advantage of using these coefficients is the possibility of increasing or decreasing the approximation level, only adding or removing the last coefficients, respectively. Likewise, this effect reflects on words, since adding or removing a letter also means increasing or decreasing the approximation level of the sequence. It is important to emphasize that the coefficients are calculated only once, and there is no need to recalculate them, if it is necessary to change the approximation level of the symbolic representation.

**Figure 9:**
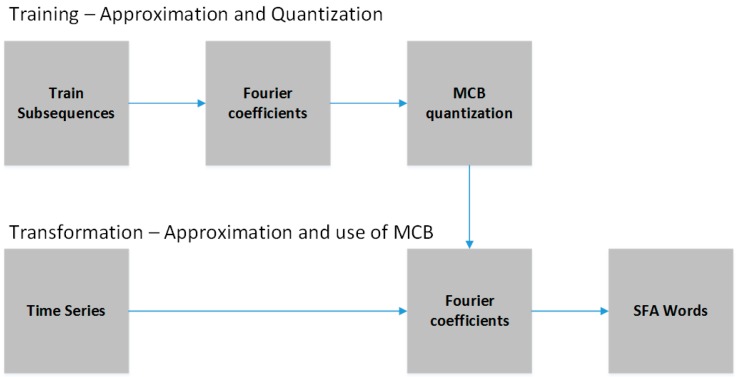
Flow of SFA steps.

Figure 9 depicts the flow of the SFA steps. As can be seen, the MCB is created based on a set of time series samples. This means that the MCB must be initially trained with sufficiently representative sequences. After the training, the MCB can be used in the process of transformation and generation of the words of the temporal series. Finally, the SFA algorithm has the following input parameters: (a) time window size; (b) data normalization around the mean and standard deviation (true or false); (c) word size, and (d) alphabet size.

## 4. Classification Approaches

The data format based on the discrete domain features has directed new studies from the perspective of adaptation and implementation of new algorithms capable of acting in the context of time series classification. In this sense, the classification algorithms based on these features use, in general, the analysis of the words frequency throughout the time series. The words frequency distribution can be obtained through a Bag of Words (BoW) or a Histogram of Words generated after discretization process.

In context of HAR, each activity has an associated set of words. The intuition is that the words distribution allows the discrimination of human activities facilitating the performance of the classification algorithms. From this perspective, this section presents two groups of algorithms, identified in the literature, adapted to act with the discrete domain features. The first group deals with vector space-based algorithms such as SAX-VSM [22] and BOSS-VS [24] and the second group deals with conventional shallow algorithms such as BOSS-Model [23] and WEASEL [25].

### 4.1. Vector Space-Based Approaches

Vector space model [34], commonly used in the information retrieval area, has been the target of several adaptations to the context of HAR in several literature works [22,23,24,25,26,27,28,29,30,31,32,33,34,35]. This approach has been widely used because of the ease for manipulating texts. In short, the vector space model is used to retrieve relevant documents from a collection based on a query composed of keywords. In the context of HAR, it is possible to make the following associations and adaptations:

1. Human activities can be translated as the collection of documents.
2. The words generated for each activity by means of the discretization process are equivalent to the words of the document body.
3. A query can be translated as the words of a non-labeled time series where the objective is to find out the level of similarity between them and the words of each activity.

Formally, the vector model is represented by vectors in space $R^{n}$, where n represents the dimension of space R. Each dimension is represented by an activity vector $A=(a_{1},a_{2},\ldots ,a_{n})$, where a represents an activity and n represents the number of activities. Each activity $a_{n}$ contains a vector of words $p=(p_{1},p_{2},\ldots ,p_{j})$, where p represents a word in position j. Each word $p_{j}$ has an associated weight $w_{i,j}$, where $w_{i,j}\geq 0$. Also, each activity $a_{n}$ is represented by a vector of weights $W_{j}=\left(w_{1,j},w_{2,j},\ldots ,w_{i,j}\right)$, where i is the quantity of all distinct words appearing in the activity $a_{n}$ and j is the vector size. The weight $W_{j}$ is calculated by taking into account the strategy tf*idf, that is, the multiplication of the words frequency, tf, by its importance, idf. The word frequency $tf_{p,A}$ is calculated according to Equation (3):

(3) $$tf_{p,A}=\;\{\begin{array}{c} \begin{array}{cc} \log\left(1+f_{p,A}\right), & se\;f_{p,A}>0 \end{array}\\ \begin{array}{cc} 0,\; & \;otherwise \end{array} \end{array}$$

where $f_{p,A}$ is the word frequency in the activity A and, $tf_{p,A}$, is the word logarithmic frequency (normalized). The importance of the word is calculated by means of the IDF (Inverse Document Frequency) that is given by Equation (4):

(4) $$idf_{p,\;T}=log\frac{T}{\left|\;\{A\in T\;:p\in A\;⋀\;f_{p,A}>0\}\right|}$$

where, $idf_{p,\;T}$ is the importance of the word p in the time series T that represents the data for all activities available (or collection of documents) and $f_{p,A}\;>0$. Therefore, tf*idf(p,A,T) of the word p in activity A is defined by Equation (5):

(5) $$tf*idf\left(p,A,T\right)=\;tf_{p,A}*\;idf_{p,T}=W_{j}$$

The weight matrix W represents the tf*idf of all activities, as shown in Table 1.

To calculate the weight vector of the query, the procedure is as follows: Calculate tf for all query words. For idf, reuse the same values of the words idf used to calculate the weight matrix.

Finally, a similarity measure is applied between the weight matrix and the query vector for discovering the closest (or similar) activity from the query. Thus, the similarity between two vectors of weights is given by Equation (6):

(6) $$similarity\left(Q,\;A\right)=\frac{\vec{Q}\cdot \vec{A}}{\left\|\vec{Q}\|\cdot \|\vec{A}\right\|}=\frac{\sum_{p\in Q}tf\left(p,\;Q\right)\;\cdot \;tfidf\left(p,\;A\right)}{\sqrt{\sum_{p\in Q}{\left(tf\left(p,\;Q\right)\right)}^{2}}\sqrt{\sum_{p\in C}{\left(tfidf\left(p,\;A\right)\right)}^{2}}}$$

For two vectors $\vec{Q}$ (query) and $\vec{A}$ (activity), the cosines similarity measure is based on the sum of the internal product between these vectors divided by the product of the query and activity vector norms. Thus, the label of the closest vector obtained by the similarity metric is assigned to the non-labeled series. In this context, this section presents two algorithms based on the vector model adapted for time series: SAX-VSM [22] and BOSS-VS [24].

#### 4.1.1. SAX-VSM—Symbolic Aggregate ApproXimation in Vector Space Model

SAX-VSM [22] is a time series classification algorithm based on SAX. This algorithm is divided into two phases: training and classification. The training phase follows the following steps:

1. Discretize the time series for each activity with the SAX algorithm. The parameters that should be chosen are the time window size, PAA size, and alphabet size.
2. Identify the distinct words and group them by activity in their respective BoW (Bag of Words) associated with their respective frequencies.
3. Calculate the weight vector of each BoW based on tf×idf.
4. Assemble the weight matrix based on the weight vectors for each activity.

Then, the classification phase follows the following steps:

5. Discretize the time series referring to the new activity with the SAX algorithm with the same parameters defined in the training stage.
6. Calculate the weight vector for the new query words generated by SAX.
7. Compare the query weight vector with the weight matrix generated in the training step using the cosine similarity formula. Classification of the weights vector of the query is based on the greater similarity with the weights vectors of the activities.

**Figure 10:**
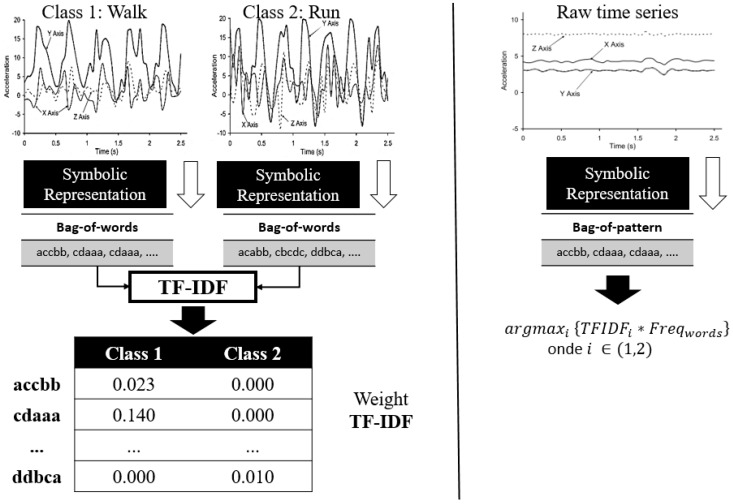
Example of the data processing steps of the SAX-VSM algorithm.

Figure 10 shows an example of the SAX-VSM training and classification steps.

#### 4.1.2. BOSS-VS—Bag-Of-SFA-Symbols in Vector Space

**Figure 11:**
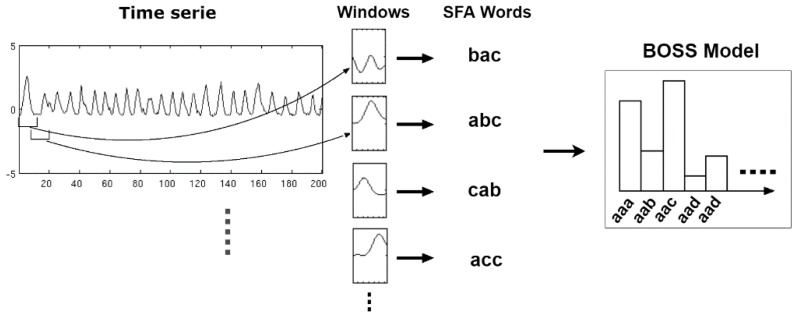
Example of discretization and histogram generation by SFA.

BOSS-VS [24] is a time series classification algorithm based on the SFA. This algorithm is also divided into the training and classification phases similar to training and classification steps from SAX-VSM described the Figure 10. The main difference between one and other is that BOSS-VS uses SFA in the discretization phase and Bag of Patterns in the data representation phase. The training phase follows the following steps:

1. Discretize the time series for each activity with the SFA algorithm. The parameters that should be chosen are time window size, word size, and alphabet size. In addition, it should be stated whether the data should be normalized or not.
2. Create the histograms for each activity. In BOSS-VS these histograms are known as BOSS (Bag-Of-SFA-Symbols). The set of all histograms is called the BOP (Bag of Patterns). The BOP generation structure can be seen in Figure 11.
3. Calculate the weight vector of the BOP based on tf×idf.
4. Assemble the weight matrix based on the weight vectors for each activity.

Then, the classification phase follows the following steps:

5. Discretize the time series regarding the new activity with the SFA algorithm with the same parameters used in the training phase.
6. Calculate the weight vector for the new query words generated by the SFA.
7. Compare the weight vector of the query with the weight matrix generated in the training step using the cosine similarity formula. Classification of the weights vector of the query is based on the greater similarity of the weight vector of the activities.

### 4.2. Approaches Based on Shallow Algorithms

Classification algorithms based on shallow machine learning are widely used to classify human activities using the time and frequency domains features. In the context of the discrete domain features, some of these algorithms were adapted to work with histograms of words. In this sense, this section presents two algorithms based on the SFA, which use the shallow algorithms 1-NN in the case of the BOSS [23] and the Logistic Regression in the case of the WEASEL [25], respectively.

#### 4.2.1. BOSS—Bag-Of-SFA-Symbols

**Figure 12:**
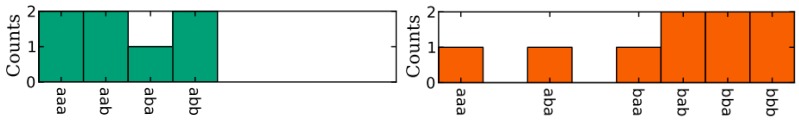
Example of two BOSS histograms.

BOSS [23] is a classification algorithm based on the analysis of the distances between histograms generated from a time series. The calculation of the distance between two histograms is based on the following principle: two sequences are similar if they share the same words with similar frequency distributions. In this case, the distance is calculated by the Equation (7):

(7) $$dist\left(BOSS_{1},BOSS_{2}\right)=\overset{n}{\underset{k=1}{\sum }}{\left(BOSS_{1}\left(f(p_{k})\right)-BOSS_{2}\left(f(p_{k})\right)\right)}^{2}{}_{BOSS_{1}\left(f(p_{k})\right)>0}$$

where $BOSS_{1\;e\;2}$ are the histograms to be compared, $f(p_{k})$ is the frequency of the word $p_{k}$ and n is the number of words in the histogram. Figure 12 shows an example of two clearly different histograms. The result of calculating the distance between them is $dist\left(BOSS_{1},BOSS_{2}\right)=9$.

The training step in the BOSS consists of generating the histograms for each activity. The classification step consists of the analysis of unlabeled histograms based on the calculation of the BOSS distance using the algorithm 1-NN.

#### 4.2.2. Weasel—Word ExtrAction for Time SEries cLassification

Weasel [25] is a classification algorithm that uses a linear strategy based on the logistic regression algorithm to classify histograms generated from a time series. Logistic regression generates models based on the scalar product between the features vectors with their respective weights. These vectors are extracted from the histograms of each activity. In other words, logistic regression generates a linear function that represents the word distribution of each activity and the weights are used to highlight the relevant features and suppress the irrelevant ones.

**Figure 13:**
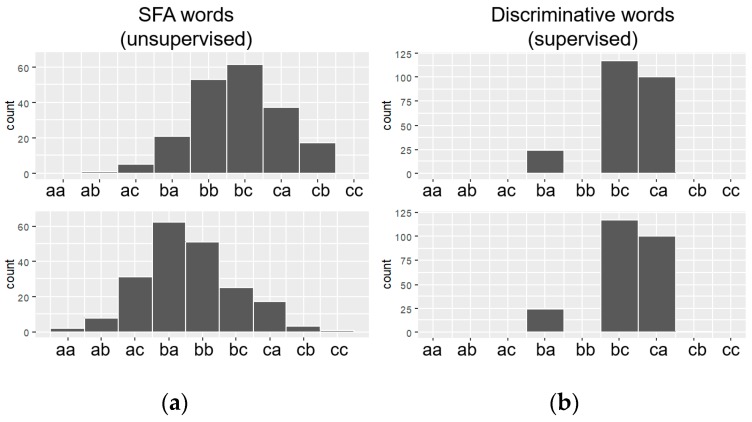
Difference between the histograms generated by the SFA (**a**) and SFA-W (**b**).

Weasel has proposed some innovations in data preparation before sending them to the logistic regression. The first innovation is related to a modification in the SFA algorithm with the purpose of generating relevant words for a certain class. This means that in addition to the discretization function, the SFA-W (modified SFA) also acts as a feature selection algorithm. From this perspective, the SFA begins to act in a supervised manner, and at the moment of generating the words, the SFA-W verifies the relevance of each word in a given class. Then, the SFA-W decides whether the word generated should be retained or discarded. Figure 13 shows an example of the difference between histograms resulting from SFA and SFA-W.

SFA-W modifies the SFA in the approximation and quantization step. In the approximation step, the first requirement is that the data must be normalized around the mean and standard deviation. Unlike the SFA that selects the first coefficients without looking at the classes, the SFA-W applies the statistical ANOVA F-test [36] to select the most representative coefficients of the DFT for each class. The reason for applying this statistical test is that the best approximation coefficients vary from data to data. The ANOVA *F*-test assumes that the data follow a normal distribution, hence the normalization requirement. In addition, the sequences used in MCB training are extracted without overlapping in order to guarantee independence between them. This strategy helps to decrease the overfitting of the classification model.

**Figure 14:**
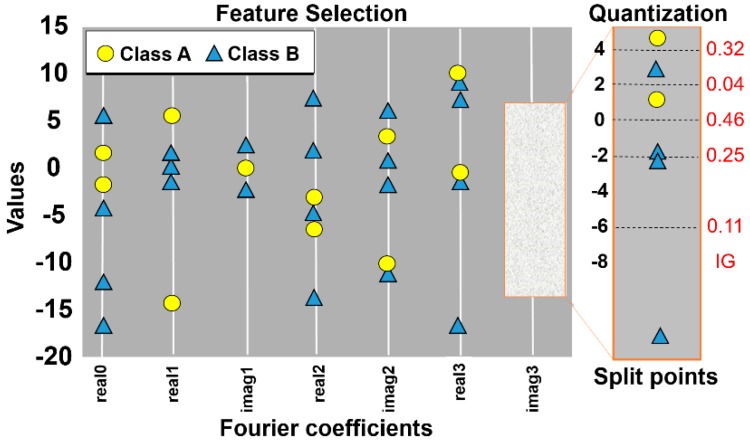
Example of how the SFA-W discretization process works. In this example, two classes are used ‘A’ and ‘B’, with the real0 and imag3 coefficients selected as the best. Then, the best cut-off point was 0.46.

In the quantization step, the columns of coefficients selected by the ANOVA F-test are divided into two partitions. The goal is that each partition separate the bins where most of the symbols associated with each of them belong to the same class. For this, the best cut-off point between one and another partition is defined by the information gain algorithm. The purpose of this algorithm is to minimize the entropy between the classes and to maximize the information gain of the symbols. At this point, words are filtered and only words that are relevant to a particular class remain. Figure 14 shows an example of how the SFA-W approximation and quantization process occurs.

The second innovation of Weasel is the addition of new useful information to the histogram. Such information consists of adding bigrams and new words representing different window sizes. Regarding to the bigram, WEASEL introduced the concept of n-grams in the features of the discrete domain. For example, if the SFA-W generates the words “aab”, “abc”, “acc” and “aaa” then the bigrams for these words are defined as “aab abc” and “acc aaa”. Therefore, the bigrams and their respective frequencies are also included in the histograms. The advantage of the bigram is the addition of new patterns that cannot be captured by a single word. On the other hand, the drawback of bigrams is the increase of the feature space. For this reason, the authors choose the logistic regression algorithm.

In an attempt to reduce the size of the feature space resulting from the use of bigrams and words of different window sizes, the Weasel applies the Pearson Chi-square test ($\chi^{2}$). The purpose of this test is to select the relevant words for the histogram of each class based on the analysis of word frequencies. This statistical test is applied over words frequencies of two histograms, if any word of observed frequency is significantly differ from the expected frequency, then this word is eliminated from the histogram. The decision to eliminate a word is based on a threshold defined by the Chi-Squared result.

**Figure 15:**
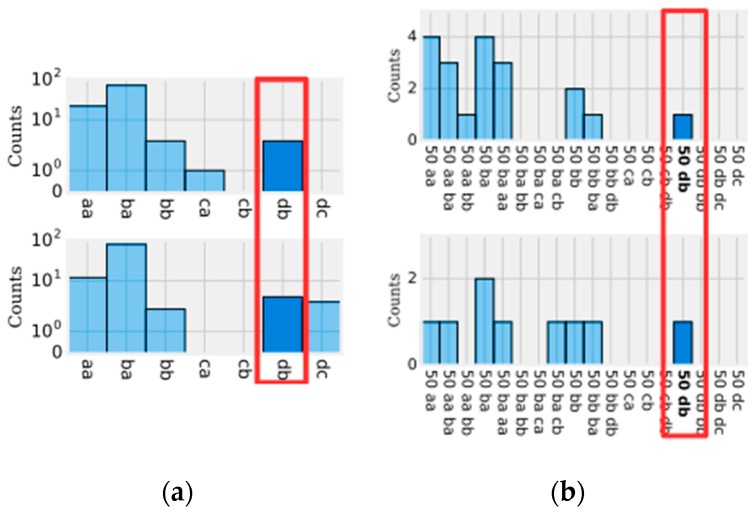

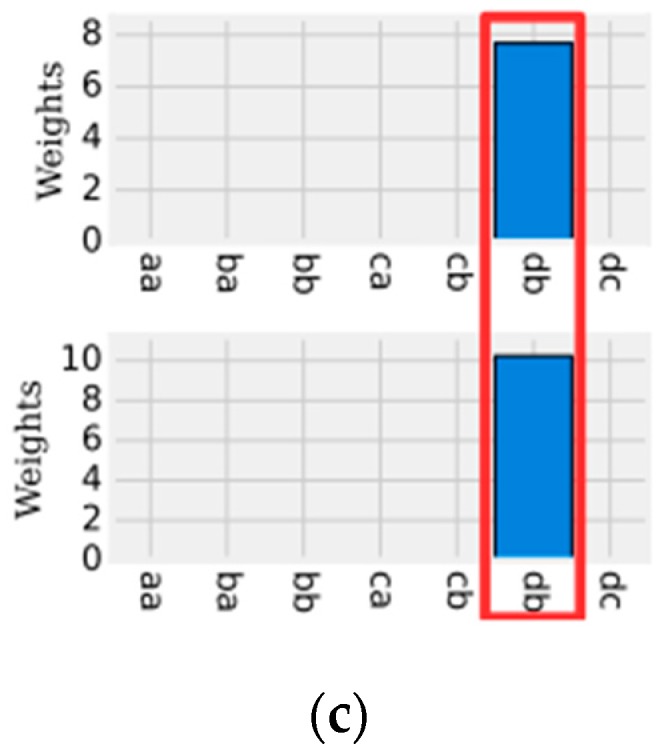
Example of the steps of histograms manipulation based on the SFA-W (**a**); bigrams (**b**); Chi-Squared (**c**). Note: the value 50 indicates the size of the window [25].

After that, the resulting histograms are used to train the logistic regression classification algorithm. Figure 15 shows an example of the histograms’ manipulation flow with the above-mentioned techniques.

## 5. Multidimensional Time Series

Classification algorithms mentioned above are prepared to handle only a single time series. The problem is that the data extracted from the inertial sensors have multiple time series, that is, the time series referring to the three-dimensional coordinates x, y and z. For this reason, it is necessary to adopt strategies capable of adapting these algorithms to act with multiple time series or multiple variables. In this sense, this work proposes two strategies.

The first strategy consists of a change in the database format so that the symbolic classification algorithms (SAX-VSM, BOSS, BOSS vs. and WEASEL) are able to stack histograms of each coordinate synchronously. The intuition of this strategy is that several segments of different synchronized time series are organized in the same tuple with the purpose of producing the stacking effect. The advantage of this approach is that there is no need to make any changes to the Bag of Patterns strategies used by the BOSS or the Bag of Words strategy used by SAX-VSM. For this reason, any of the algorithms described in this chapter can be used. The data entry format consists of the following matrix organization of the x, y and z coordinates:

$$x_{1j},\ldots ,x_{\left(i+k\right)j},y_{1j},\ldots ,y_{\left(i+k\right)j},z_{1j},\ldots ,z_{\left(i+k\right)j}$$

$$x_{2j},\ldots ,x_{\left(i+k\right)j},y_{2j},\ldots ,y_{\left(i+k\right)j},z_{2j},\ldots ,z_{\left(i+k\right)j}$$

$$x_{ij},\ldots ,x_{\left(i+k\right)j},y_{ij},\ldots ,y_{\left(i+k\right)j},z_{ij},\ldots ,z_{\left(i+k\right)j}$$

where $x_{ij},\;y_{ij},\;z_{ij}$ represents the time series for each coordinate, i represents the position of the time series values, k represents window size and j represents the position of the column. In summary, the matrix means that x, y and z are split into same-size segments, using windowing for each coordinate axis. After, the segments are concatenated with one another and the stacking works by virtue of the algorithms using the same window size k, such that words will be computed independently for x, y and z. Thus, this strategy avoids extra processing related to isolated discretization of each time series so that words are later stacked in a single histogram.

The second strategy consists of the use of data fusion techniques such as magnitude and PCA (Principal Component Analysis) for transforming the multidimensional time series into a one-dimensional time series to use the existing symbolic representation algorithms in their natural form. In the case of magnitude, the formula used to transform the coordinates is based on Equation (8):

(8) $$M\left(X_{i}\right)=\sqrt{x^{2}{}_{i}+\;y^{2}{}_{i}+\;z^{2}{}_{i}}$$

where, x, y and z are the coordinates of the inertial sensors and $X_{i}$ is the data sequence. In the case of PCA, only the first component is considered since it contains the main information of the components generated.

In addition, Schäfer and Leser [37] proposed an algorithm based on the stacking of words called Weasel + MUSE. This algorithm presents a differential with the application of the derivation technique [38] of histograms. This technique consists of relating the histograms of each coordinate by calculating the difference between the neighboring histograms of each dimension. Weasel + MUSE uses the Weasel framework, i.e., all the advantages and disadvantages of using bigrams, window size, and feature selection are incorporated into Weasel + MUSE.

## 6. Experiments and Results

This section presents the experimental protocol and the results concerning the comparative analyzes between the shallow algorithms and the symbolic representation algorithms. The experimental protocol presents a description of the databases used in the experiments. After, it describes the baselines with emphasis on the presentation of the time and frequency domain features. Then, the configurations and parameters used in the algorithms are presented. Finally, the results address a comparative analysis involving the study of the solutions in terms of precision, processing time, and memory consumption.

### 6.1. Databases Used in Experiments

The UCI-HAR database [26] was collected with data from 30 people aged 19–48 years. Each person performed 6 physical activities such as walking, sitting, standing, lying down, and going up and down stairs. The data were collected from a Samsung Galaxy S2 handset using the accelerometer and gyroscope sensors at a frequency of 50 Hz. The collection was performed with the smartphone located at the user’s waist. All steps of data collection were recorded and the data was manually labeled. UCI-HAR has 1,311,439 samples.

The Shoaib database [28] was collected with data from 10 male users ranging in age from 25 to 30 years. Each user performed eight activities for 3 to 4 min for each activity. Such activities include walking, running, sitting, standing, jogging slowly, biking, climbing stairs and coming down stairs. Data were collected from a Samsung Galaxy S2 (i9100) mobile phone using the accelerometer, linear accelerometer, gyroscope, and magnetometer sensors at a frequency of 50 Hz. Users were equipped with five smartphones located in five body positions including right and left pocket of the pants, waist, wrist and forearm. Shoaib has 629,977 samples.

The Wireless Sensor Data Mining (WISDM) database [27] was collected with data from 36 users. Each user performed 6 activities such as walking, sitting, standing, lying down, and going up and down stairs. Data was collected from an Android smartphone (Nexus One, HTC Hero) at a frequency of 20 Hz. The collection was carried out with the smartphone located in the front pocket of the user’s pants. WISDM has 1,098,830 samples.

### 6.2. Baselines

The baselines used for comparison with the symbolic representation algorithms are the shallow algorithms including the Decision Tree, Naive Bayes, KNN (k-Nearest Neighbors) and the SVN (Support Vector Machine) commonly used in the HAR literature [39,40,41,42]. It is not the purpose of this paper to provide theoretical information on how each of these algorithms work; more details on each of them can be found in [43]. The deep algorithms were not included in the experiments because of the high processing costs and the difficulty of implementing a solution completely dependent on the smartphone.

Shallow algorithms are trained based on the time and frequency domain features listed in Table 2. Time domain features are represented by mathematical functions capable of extracting statistical information from the signal. On the other hand, the frequency domain features present an alternative of signal analysis based on the frequency spectrum of the values of a certain time window. The mathematical functions commonly used in this context are Fast Fourier Transform (FFT) [44] or Wavelet [45]. Then, the frequency features listed in Table 2 are extracted based on the FFT or Wavelet results.

In the case of the inertial sensors, the features are extracted after the data segmentation step. This step consists of segmenting the sensor signal into time windows represented by a sequence $X=\left\{x_{p},x_{p+1},\ldots ,x_{p+w-1}\right\}$, where w represents the window size and p represents an arbitrary position, such as 1≤p≤n−w+1, where n represents the sequence size. In addition, time windows may overlap each other, that is, two time windows may have intersections between them. The purpose of the overlap is to minimize the number of time windows that contain different label data. Ideally, all windows have consistent data, that is, only one label. Finally, the feature extraction step consists of a data transformation process performed on top of the segmented data.

The treatment of multidimensional time series using the shallow classification algorithms based on the time and frequency domain features is simpler. This process occurs in the feature extraction step by concatenating the feature vectors of each coordinate. The result is a single database with the features of all coordinates.

### 6.3. Algorithm Settings and Parameters

This section presents the settings and parameters used by baselines and by the symbolic representation algorithms. A common configuration to all of them is related to the classification models evaluation strategy. In this case, the strategy adopted was cross-validation 10-folds. Each fold contains random data belonging to all users. In addition, the evaluation metric used is the accuracy. To simplify the presentation of the results, the experiments were performed only with the inertial sensor accelerometer data. According to Sousa et al. [9] this sensor is sufficient to represent the physical activities of users. In the others, the configurations used specifically for baselines are based on results of experiments carried out in the literature [3,9,46], as: (1) time window size: 5 s; (2) overlap: 50%; (3) features: time domain, frequency domain derived from FFT, and Wavelet.

In the case of the symbolic representation algorithms, this work presents the results of the multidimensional time series manipulation strategies explained in Section 6. In addition, the parameters that must be defined are: window size, word size, and alphabet size. Some parameters were defined based on suggestions from the literature of the symbolic representation algorithms [16,17]. The other parameters were defined based on experiments done with the data extracted from the inertial sensors. These parameters were evaluated in the UCI-HAR database and replicated to the other databases. Thus, the configurations defined were:

- Window size: algorithms were evaluated with different window sizes ranging from 20 to 250 samples. For each database, the best time window size found was equal to the number of samples contained in 1 s, that is, the value equivalent to the frequency rate of the database collection.
- Word size: algorithms were evaluated with different word sizes ranging from 4 to 8 letters. These sizes were chosen because they are commonly used by SAX and SFA authors. In this case, it was observed that, the larger the word size, the better the accuracy of the classification models. This occurs because larger words tend to get closer to the original sequence. Therefore, the word size that was defined was 8 letters.
- Alphabet size: The alphabet size was set to 4 characters, as recommended by the authors of the SAX algorithm [16] and SFA [17].

Experiments were run on an Intel Core i7-3612QM CPU 2.10 GHz computer equipped with 8.00 GB of RAM. The time-based experiments were run three times and averaged over time.

### 6.4. Results

This section presents a comparative analysis of the symbolic representation algorithms SAX-VSM, BOSS, BOSS vs. and WEASEL with the Decision Tree, Naive Bayes, KNN and SVN shallow algorithms combined with the time and frequency domain features. Results are divided into three scenarios. The first deals with the evaluation of the classification models accuracy. The second deals with the evaluation of the processing time in the steps of feature extraction and the classification models training. Finally, the third deals with the evaluation of the space consumption (memory) for each one of these steps.

#### 6.4.1. Evaluation of the Precision of Classification Models

This section presents the results regarding the accuracy of classification models. Table 3 and Table 4 present a summary of the results grouped into six categories. The first category deals with the classification models generated by shallow algorithms trained with the time domain features. The second and third categories deal with the classification models generated with frequency domain features based on FFT and Wavelet. The fourth category deals with the classification models generated by the symbolic representation algorithms using the coordinate fusion strategy based on the histograms stacking. Finally, the fifth and sixth categories deal with the generation of classification models using the coordinate fusion strategies based on magnitude and the PCA.

In the context of shallow algorithms, the results show that classifiers based on time domain features are superior in terms of accuracy compared to classifiers based on frequency domain features. In this case, the highlight should be given to the Decision Tree and KNN classifiers, since both have an average accuracy rate of 89.19% and 90.77%, respectively, considering all the databases evaluated. On the other hand, the accuracy rates of the same classifiers based on frequency domain features derived from FFT and Wavelet, decreased by an average of 22.31% when compared to the results of time domain features. In addition, among all classifiers, the worst performance is Naive Bayes with an average accuracy below 66.15% for the time domain features.

These results are not new; Sousa et al. [9], for example, reached the same conclusion and they affirm that time domain features are sufficient to represent activities from inertial sensors’ data, especially using algorithms of the decision tree family where they have a lower processing cost compared to the KNN and SVM.

In the context of the symbolic representation algorithms, the results show that all the coordinate fusion strategies using the symbolic representation algorithms BOSS and BOSS vs. offer the best results in terms of the classification models precision. In particular, the BOSS algorithm obtained an accuracy rate of 100% for all databases evaluated. This may have been caused by overfit of the parameters setting that allowed the BOSS model to suffer overfitting. The BOSS vs. classifier obtained an average accuracy rate of 97.9% confirming the overfit of the BOSS algorithm. On the other hand, the WEASEL algorithm obtained the worst accuracy with an average rate for all databases of 50.75%. This shows that Weasel is not suitable for use in combination with coordinate fusion strategies.

As can be observed, the symbolic representation algorithms obtained the best accuracy when compared to the shallow algorithms combined with the time domain features. Therefore, this proves the efficiency of these algorithms in the context of HAR in terms of the classification models’ accuracy. The next sections show the efficiency of these algorithms in terms of processing time and memory consumption.

#### 6.4.2. Processing Time Evaluation

This section presents the results regarding the processing time of the steps feature extraction and training of classification model in the context of shallow and symbolic representation algorithms.

Table 5 shows the processing times of the feature extraction steps of the time and frequency domains and the discrete domain using the SAX, SFA and SFA-W discretization algorithms. As can be seen, the average processing time of the time and frequency domain features for the WISDM database with 1,098,830 samples is 12,461.33 milliseconds. While the average for the discretization algorithms is 1892.66 milliseconds. This represents a reduction of 84.81% considering the discrete domain in relation to the other feature domains.

In particular, SFA is the fastest algorithm (with 418 milliseconds) because SFA is optimized to treat words as a set of bits. For example, an alphabet of size 4 is represented by only 2 bits, i.e., if the alphabet letters are ‘a’, ’b’, ‘c’ and ’d’, then the representation of each letter will be ‘a = 00’, ‘b = 10’, ’c = 11’, ‘d = 01’. In this way, the processing time gain reaches 96.28% in relation to the other features domains. On the other hand, the SAX algorithm manipulates words without any optimization, and yet the processing time is 2636 milliseconds. The processing time of the SFA-W is equivalent to SAX due to the selection process of important words with the application of ANOVA Test and information gain.

Table 6 shows the processing time of training of classification models by shallow classifiers and symbolic representation classifiers. As can be seen, the results can be divided into two categories. The first category called ‘A’ involves the results of the classifiers (Naive Bayes, KNN, SAX-VSM and BOSS), considering the time threshold below 151 milliseconds. 

The second category called ‘B’ involves the results of the classifiers (Decision Tree, SVM and BOSS VS), considering the time threshold above 925 milliseconds. We consider that WEASEL does not fall into the categories ‘A’ and ‘B’ because of the longer processing time. On average, the classifiers of the category ‘A’ are 80.40% faster than the Weasel and the classifiers of the category ‘B’ are 99.62% faster than the Weasel. The reason for the Weasel to be the slowest classifier is because the histograms are composed of unigrams, bigrams, and words from different window sizes. In addition, Weasel applies the Chi-Squared statistical test and uses a logistic regression to generate the model, as described in Section 4.2.2.

Classifiers of category ‘A’, Naive Bayes, KNN, SAX-VSM and BOSS, are the ones that act faster in the step of the classification models’ training. The average processing time for these algorithms is 70.17 milliseconds. This means that these algorithms are 52 times faster than the average processing of classifiers of category ‘B’. It is important to emphasize that although some algorithms are faster in the training of the classification models step, they are not necessarily faster in the classification step. However, what happens in practice is the classification of only 1 instance over time and, therefore, it is insignificant to evaluate the processing time in this context.

In this sense, we conclude that the BOSS is the fastest algorithm since the sum of the processing time of both steps for the WISDM database, for example, is 464 milliseconds. The second fastest algorithm is the SAX-VSM with a sum of 2675 milliseconds. In addition, it is important to note that coordinate fusion strategies based on magnitude and PCA are those that require a lower cost of data processing compared to the histogram stacking strategy. More specifically, the strategy based on signal magnitude is the one that consumes fewer computational resources.

#### 6.4.3. Memory Consumption Evaluation

This section presents the results regarding memory consumption before and after the application of the feature extraction step for the UCI-HAR, WISDM and SHOAIB databases. The results are described in Table 7 as the unit of measurement Bytes. The strategy used to collect this data was as follows. The first step consists of calculating the number of Bytes occupied by raw data in RAM memory. The second step consists of applying the feature extraction process. The third step is calculating the number of Bytes occupied by processed data in RAM memory. For the frequency domain only the features derived from the FFT were chosen, since the features derived from Wavelet have similar results. For the discrete domain, the SAX algorithm was chosen to discretize the database.

As can be seen, the time features can reduce the original data by 73.90%, on average, for all databases. For example, UCI-HAR data has been reduced from around 43 million bytes to about 9 million bytes. For frequency features the data reduction rose to 92.66%. The reason is that the number of frequency features is 68.96% smaller compared to the time features, i.e., the experiments were performed with 29 time features and nine frequency features as shown in Table 2. On the other hand, the discrete features reduce, on average, the memory space by 94.48%, a growth of 1.92% in relation to the frequency features.

## 7. Related Work

In general, the literature presents two groups of discretization algorithms [17]. The first group deals with the numerical approximation algorithms including Discrete Fourier Transform (DFT) [45], Discrete Wavelet Transform (DWT) [45], Chebyshev Polynomials (CP) [47], Piecewise Linear Approximation (PLA) [48], Piecewise Aggregate Approximation (PAA) [32] and Adaptive Piecewise Constant Approximation (APCA) [49]. The second group deals with symbolic representation algorithms including Symbolic Aggregate Approximation (SAX) [16] and Symbolic Fourier Approximation (SFA) [17]. Specifically, this research focuses on the algorithms of the second group for two reasons. The first reason is that these algorithms are considered state of the art of symbolic representation algorithms. The second reason is that all the algorithms of the second group are based on some algorithm of the first group. For example, SAX and SFA define the symbol patterns based on the PAA and the DFT coefficients, respectively.

Symbolic representation algorithms have been used to recognize recurrent (Motifs) [16] or anomalous (Discords) [30,50] patterns in time series. In the context of HAR, there are few works which use the symbolic representation algorithms [14,51,52]. Figo et al. [14], for example, use the SAX algorithm to discretize the time series. The activities classification process occurs by calculating the distance between the symbols. In this case, they used the Euclidean distances, Levenshtein [53] and Dynamic Time Warping (DTW) [54]. In the experiments a comparative analysis was performed between discrete features and time features (e.g., mean, variance, and standard deviation) and frequency features (e.g., entropy and energy). The data used were obtained from the accelerometer sensor and the activities evaluated were jump, run, and walk.

Siirtola et al. [51] proposed a new method called SAXS (Symbolic Aggregate approximation Similarity). SAXS consists of defining prototypes for each activity, so that each of them is represented by a generic word called a template. The activities classification process occurs by calculating the similarity between a new non-labeled word and the templates of each activity. Thus, the new word is sorted based on the closest template, i.e., with the most similarity. In the experiments, a comparative analysis was performed between the features extracted with SAXS and some time features (mean, standard deviation, median, quartiles, minimum, and maximum) and frequency features (sum of coefficients and zero crossing). The data used were obtained and five public databases and activities evaluated were of movement (e.g., walking, running), gestures and swimming. The evaluated classifiers were the decision tree, KNN and Naive Bayes. The results show that SAXS is capable of increasing accuracy by 3% and by up to 10% if combined with the discrete features and the time and frequency features.

Terzi et al. [52] also use SAX to extract the discrete domain features. In the classification of activities, the solution uses an adaptation of the k-NN to verify the minimum distance between labeled and non-labeled words. Finally, an extension of the SAX is proposed for manipulating multidimensional time series. In this case, what occurs is a linear combination of the minimum distances for each coordinate. The experiments were carried out in the UCI HAR database and evaluated the activities walking, and climbing and descending stairs. The results show accuracies of 99.54% and 97.07% for the respective classes of activities.

## 8. Conclusions

This work introduced and evaluated two algorithms for data symbolic representation, SAX and SFA, in the context of HAR based on smartphones instrumented with inertial sensors. These algorithms act in the feature extraction step and are responsible for generating features belonging to the discrete domain. The advantages of using symbolic representation algorithms include reducing dimensionality and numerosity of the data. In addition, these algorithms make two steps of traditional HAR methodology obsolete. The first step deals with the data noise reduction and the second step deals with the feature selection. In addition, this work evaluated some classification algorithms adapted to manipulate symbolic data, such as SAX-VSM, BOSS, BOSS-VS and WEASEL. In addition, this work proposed two strategies to manipulate multidimensional time.

Experiments were performed in three UCI-HAR, SHOAIB and WISDM databases and the results show the efficiency of the strategy based on symbolic representation algorithms in three perspectives: classification model accuracy, processing time of feature extraction, processing time of classification model training and memory consumption. The main conclusions drawn from these algorithms are:

(1) The BOSS and BOSS vs. algorithms obtained the best accuracy rates using the three coordinate fusion strategies (histogram stacking, magnitude, and PCA).
(2) The Weasel algorithm did not perform well with coordinate fusion strategies.
(3) The merging strategy based on coordinate histogram stacking has generated the best classifiers.
(4) Processing time in the feature extraction step is faster with discrete domain features. In average, discrete features are 84.81% faster compared to the time and frequency features. SFA is the fastest method taking an average of 418 milliseconds for a database with amount of data around 1 million samples (WIDSM).
(5) Extraction of discrete domain features is rapid using SFA due to the optimization process based on the data transformation for bits.
(6) SAX-VSM and BOSS algorithms are the fastest algorithms in the feature extraction and training classification models steps, surpassing all the baselines. On the other hand, the BOSS vs. and Weasel algorithms are the slowest algorithms in the training classification models step.
(7) Discrete domain features reduce memory consumption by 94.48% compared to the frequency features with 92.66% and time features with 73.90%. Although the space memory difference between frequency features and the discrete features is only 1.92%, the frequency features lose in terms of classification model accuracy and processing time.

Therefore, it is concluded that strategies based on symbolic representation algorithms are able to reduce the computational cost of HAR solutions, enabling their implementation in smartphones with limited memory and processing resources.

## Figures and Tables

**Table 1:** Example of the weight matrix generated by TF-IDF.

	Activity 1	Activity 2
accbb	0.023	0.0
cdaaa	0.14	0.0
...	...	...
ddbca	0.0	0.010

**Table 2:** List of time and frequency domains features.

Domain	Features
Time	min, max, amplitude, amplitude peak, sum, absolute sum, Euclidian norm, mean, absolute mean, mean square, mean absolute deviation, sum square error, variance, standard deviation, Pearson coefficient, zero crossing rate, correlation, cross-correlation, auto-correlation, skewness, kurtosis, area, absolute area, signal magnitude mean, absolute signal magnitude mean, magnitude difference function.
Frequency	Energy, energy normalized, power, centroid, entropy, DC component, peak, coefficient sum.

**Table 3:** Results regarding the accuracy of the classification models generated by the shallow algorithms combined with the time and frequency domain features.

Features	Algorithm	UCI-HAR	SHOAIB	WISDM
Time Features	Decision Tree	84.64	94.56	88.38
	Naive Bayes	57.36	73.74	67.34
	SVM	81.78	92.04	83
	KNN	87.83	96.76	87.72
FFT	Decision Tree	69.48	76.02	68.05
	Naive Bayes	27.81	57.39	35.03
	SVM	60.12	70.76	63.51
	KNN	65.84	74.08	65.94
Wavelet	Decision Tree	74.96	79.22	77.85
	Naive Bayes	25.78	69	45.95
	SVM	60.17	68.22	72.58
	KNN	61.03	63.72	73.53

**Table 4:** Results regarding the accuracy of the classification models generated by the symbolic representation algorithms using the coordinate fusion strategies for histogram stacking, magnitude, and PCA.

Features	Algorithm	UCI-HAR	SHOAIB	WISDM
Axis Fusion	SAX-VSM	95.7	91.3	75.1
	BOSS	100	100	100
	BOSS-VS	98.5	99.9	94.4
	WEASEL	52.9	48.4	59.9
Magnitude	SAX-VSM	87.7	81.7	60.8
	BOSS	100	100	100
	BOSS-VS	98.9	99.6	96
	WEASEL	36.6	54.09	57.9
PCA	SAX-VSM	69.6	80.7	36.5
	BOSS	100	100	100
	BOSS-VS	96.9	99.7	97.2
	WEASEL	41	43.6	62.4

**Table 5:** List of processing time for feature extraction step in time, frequency, and discrete domain.

Feature Extraction Time (ms)			
Features	UCI-HAR	SHOAIB	WISDM
Time Features	14,458	6434	11,240
Frequency Features (FFT)	17,008	8227	17,750
Frequency Features (Wavelet)	13,519	5387	8394
SAX	3121	1706	2636
SFA	559	301	418
SFA-W	4230	2858	2624

**Table 6:** List of processing time in the training step of the classification model.

Train Time (ms)			
Algorithm	UCI-HAR	SHOAIB	WISDM
Decision Tree	2534	925	2507
Naive Bayes	151	66	125
SVM	4073	1277	9233
KNN	51	45	49
SAX-VSM	76	97	39
BOSS	62	35	46
BOSS-VS	8895	2014	1976
WEASEL	31,036	7531	18,318

**Table 7:** Memory consumption in data bytes before and after the feature extraction process.

Dataset	All	Time Features	Frequency Features (FFT)	Discrete Features (SAX)
UCI-HAR	43,409,405	9,014,107	2,503,597	1,779,486
WISDM	22,035,537	7,019,442	1,967,915	866,972
SHOAIB	16,175,747	4,151,907	1,182,459	1,377,616
